# Identification of a novel ceRNA network related to prognosis and immunity in HNSCC based on integrated bioinformatic investigation

**DOI:** 10.1038/s41598-022-21473-0

**Published:** 2022-10-20

**Authors:** Hongbo Liu, Guoli Hei, Lu Zhang, Yanxia Jiang, Haijun Lu

**Affiliations:** 1 Department of Radiation Oncology, the Affiliated Hospital of Medical College Qingdao University, Qingdao, China; 2 Department of Pathology, the Affiliated Hospital of Medical College Qingdao University, Qingdao, China

**Keywords:** Cancer, Computational biology and bioinformatics, Immunology

## Abstract

Head and neck squamous cell carcinoma (HNSCC) is characterized by an immunosuppression environment and necessitates the development of new immunotherapy response predictors. The study aimed to build a prognosis-related competing endogenous RNA (ceRNA) network based on immune-related genes (IRGs) and analyze its immunological signatures. Differentially expressed IRGs were identified by bioinformatics analysis with Gene Expression Omnibus (GEO), The Cancer Genome Atlas (TCGA) and ImmPort databases. Finally, via upstream prognosis-related microRNAs (miRNAs) and long noncoding RNAs (lncRNAs) prediction and co-expression analysis, we built an immune-related ceRNA network (LINC00052/hsa-miR-148a-3p/PLAU) related to HNSCC patient prognosis. CIBERSORT analysis demonstrated that there were substantial differences in 11 infiltrating immune cells in HNSCC, and PLAU was closely correlated with 10 type cells, including T cells CD8+ (R =  − 0.329), T cells follicular helper (R = − 0.342) and macrophage M0 (R = 0.278). Methylation and Tumor Immune Dysfunction and Exclusion (TIDE) analyses revealed that PLAU upregulation was most likely caused by hypomethylation and that high PLAU expression may be associated with tumor immune evasion in HNSCC, respectively.

## Introduction

Head and neck squamous cell carcinoma (HNSCC) is a malignant tumor that arises in the mucosal linings of the oral cavity, pharynx and larynx^1^. Of all malignancies, it has the greatest incidence and mortality rates. Despite treatment with adjuvant and combination therapies, HNSCC still has a poor prognosis, with a response rate of less than 50%. Over the past decade, elucidation of the molecular and genetic landscape of HNSCC has provided novel avenues for immunotherapy treatment, and more effective early detection and treatment methods for HNSCC are urgently needed.

Competing endogenous RNAs (ceRNAs) are essentially endogenous RNAs that compete for shared microRNAs (miRNAs) to disrupt each other’s functions and thus promote the occurrence and development of multiple cancers, as proven by increasing bioinformatics and cell biology evidence^2,3^. Endogenous RNAs include long noncoding RNAs (lncRNAs), circular RNAs (circRNAs), and pseudogenes, while miRNAs act as “sponges”^4^. The abundance, remarkable tissue specificity, and association with aberrant expression landscapes of many tumor lncRNAs, which do not have protein coding potential, have been proven in numerous studies, suggesting the promising potential of such lncRNAs as diagnostic or prognostic biomarkers for diverse malignancies^5^. Research has reported that lncRNAs such as HOTAIR, UCA1, LET, MEG3, MALAT1, H19, and NAG7 are dysregulated in HNSCC^6^.

It is worth noting that the tumor microenvironment (TME) is an extensive network of multiple factors, which include invading immune cells, epithelial cells, vascular and lymphatic channels and extracellular matrix molecules. Different types of cancer have different proportions of infiltrating immune cells, which affects immunological response and prognosis^7^. Recent research has shown that ceRNA networks participate in tumor cell-immune system communication, and several molecular signatures are biomarkers for this crosstalk^8,9^. These characteristics may help predict responses to different therapies, especially immune checkpoint blockade treatments targeting the coinhibitory receptor programmed death-1 (PD-1)/programmed death-ligand 1 (PD-L1), which have revolutionized cancer therapy for the past few years. The TME also affects the degree of the immune checkpoint inhibition response in HNSCC^10^. Previous research has linked the expression of PD-L1 to immune infiltrating lymphocytes and a better prognosis in patients with human papillomavirus (HPV)-negative HNSCC^11^. Despite this relationship, a large number of patients do not benefit from treatment but do experience its negative effects. Therefore, further study of immune-related targets and ceRNA networks in HNSCC is needed.

In this study, we first searched The Cancer Genome Atlas (TCGA), Gene Expression Omnibus (GEO) and ImmPort databases and then took obtained immune-related genes (IRGs) that were differentially expressed from the overlapping results. Next, through protein–protein interaction (PPI) analysis, we identified and visualized 18 hub genes. Two upregulated genes and four downregulated genes were further subjected to expression and prognostic analyses. Upstream miRNAs were predicted by TarBase and upstream lncRNAs of candidate miRNAs were predicted by miRNet. Both sets of predictions were validated through starBase. Based on the above analyses, a ceRNA network was proposed. Then, using coexpression and survival analyses, a novel immune-related ceRNA network (LINC00052/hsa-miR-148a-3p/PLAU) was identified. Finally, the methylation analysis, immune infiltration analysis, and tumor immune dysfunction and exclusion (TIDE) analysis were performed to study the methylation levels, immune infiltration levels and different immune response implications of the key genes, respectively. The factors identified in this study may be diagnostic biomarkers and immunotherapy targets for HNSCC.

## Materials and methods

### Transcript collection and preprocessing

Transcriptome profiles (level 3), including HTseq-count and FPKM data from 546 HNSCC samples, were obtained from the TCGA database (https://cancergenome.nih.gov/). Furthermore, we extracted HNSCC-related datasets deposited up to December 2021 from the GEO public microarray database (http://www.ncbi.nlm.nih.gov/geo/). Each GEO dataset met the following requirements: (1) the following keywords were included in the search terms: (head OR neck) AND (tumor OR cancer OR carcinoma OR neoplasm); (2) the study included both an HNSCC group and a control group; and (3) the number of participants in each group was more than three. Ultimately, only the GSE6631 dataset, which includes miRNA microarray data from 22 HNSCC tissues and 22 normal tissues, was employed for analysis. All data were downloaded from public databases by “TCGAbiolinks”^12^ (R package version 2.24.3) and “GEOquery”^13^ (R package version 2.64.2) in R software. Because TCGA is a gene sequencing database financed by the United States, with enormous sample sizes, while GEO collects high-throughput sequencing data from research institutions around the world, there is a significant sample size disparity between the two databases. Most of the experimental samples submitted to he GEO database are small due to technical and financial constraints.

### Analysis of DEGs and identification of IRGs

The Ensembl database (http://asia.ensembl.org/index.html) and the “Bioconductor” package (version 3.15) in R software were used to annotate mRNA. The "DESeq2" package (version 1.36.0)^14^ in R software was used to conduct normalization and gene expression differences analysis of mRNA in the TCGA database, while “limma-voom” package (version 3.52.3)^15^ was used to standardize and differential analysis mRNA in the GEO database between HNSCC and normal tissues. Eligible differentially expressed genes (DEGs) were filtered with these criteria: *adjusted* p value < 0.05 and |log2 (Fold Change)| > 1. The DEGs were visualized with heatmaps and volcano plots generated with the R package “ggplot2” (version 3.3.6) and “pheatmap” (version 1.0.12). The integrated DEGs were screened using the Robust Rank Aggreg (RRA) package (version 1.2)^16^ in R with P value < 0.05 and |log (Fold Change)| > 1. We also used the ImmPort database (https://www.immport.org/) to identify genes strongly linked to immune activity. The four datasets were merged to yield the final list of target genes (IRGs).

### Functional enrichment analysis

To investigate the biological function of IRGs, we performed Gene Ontology (GO) and Kyoto Encyclopedia of Genes and Genomes (KEGG) enrichment analyses via the “clusterProfiler” package (version 4.4.4) and “enrichplot” package (version 1.16.2). We also presented the most highly enriched GO keywords and KEGG pathways through "ggplot2" package (version 3.3.6). Both p- and q-values < 0.05 were considered to indicate significant enrichment^17,18^.

### Hub IRG identification and survival analysis

A network of protein–protein interaction (PPI) was created using the STRING online database (http://string-db.org, version 11.3), which is widely used to investigate the interactions among proteins. Then, we utilized CytoHubba, a key node-exploring app in Cytoscape (https://cytoscape.org/, version 3.7.2), to reveal the hub genes according to their connection degrees. GEPIA2 (http://gepia2.cancer-pku.cn/), a database containing the expression and survival data of 519 HNSCC samples and 44 nontumor samples, was used to quantify IRG expression and prognostic significance. The survival information was also verified through the Kaplan–Meier (KM) plotter database (http://kmplot.com/), with p < 0.05 indicating a significant difference. Through these steps, the eligible IRGs were confirmed for further research.

### Construction and evaluation of the ceRNA network

Firstly, we identified differentially expressed miRNAs (DEMs) and lncRNAs (DELs), which generated a volcano map with adjusted P value < 0.05 and |log (Fold Change)| > 0.5^19,20^. To explore the potential mRNA–miRNA pairs, the TarBase v.8 databases (https://dianalab.e-ce.uth.gr/) were used to find the upstream miRNAs of the qualified IRGs. As potential miRNAs, only those DEMs with significant supporting data, such as western blotting, luciferase reporter assay and qPCR test data, were chosen. Then, using the starBase database (http://starbase.sysu.edu.cn/index.php) and KM plotter database, we assessed eligible miRNAs with the appropriate expression and prognostic characteristics (p < 0.05). A widely known tool for predicting miRNA targets, the miRNet database (https://www.mirnet.ca/), was utilized to suggest potential targets for lncRNAs, and the starBase database was used to confirm DELs. Finally, the lncRNA–miRNA–mRNA axis results were visualized using a Sankey diagram based on the interactions between them. Moreover, we used coexpression analysis to verify the relationship and validate the connectivity between ceRNA pairs. The complexities of the ceRNA network were visualized with Figdraw (https://www.figdraw.com). Furthermore, we assessed the prognostic 2-, 4- and 6-year predictive value of risk score based on the hub mutant genes by Receiver Operating Characteristic (ROC) curve^21^ which was generated by “survival” package (version 3.3.1) and “pROC” package (version 1.18.0), respectively.

### Estimation of the levels of infiltrating immune cells in HNSCC

Using the CIBERSORT method^22^, we analyzed 22 types of immune cells to determine their proportions in infiltrating immune cells in HNSCC. We also measured the levels in tumor and tumor-adjacent tissues separately to determine whether there were any relationships. Only samples with a CIBERSORT result of p < 0.05 were regarded as having clinical significance. The immune cell distribution and correlation in HNSCC were visualized using a violin plot and a correlation plot. TIMER 2.0 (http://timer.comp-genomics.org/), an online tool for evaluating and visualizing levels of immune infiltration in diverse malignancies, was used to analyze the prognosis signatures correlated with immune cells.

### Methylation and expression analyses of PLAU

Through the TCGA database, we compared the levels of DNA methyltransferase (DNMT1, DNMT3A, and DNMT3B) expression across individuals with high and low PLAU expression. We further explore the methylation levels of PLAU between HNSCC and normal tissues by using UALCAN (http://ualcan.path.uab.edu/). Finally, we used MEXPRESS (https://mexpress.be) to evaluate the relationship between PLAU gene expression and its DNA methylation status.

### Analysis of the relationship between PLAU and immune components

First, to evaluate the relative abundances of 22 types of immune cells in high and low PLAU expression groups, we performed immune cell subset deconvolution by using the CIBERSORT. Additionally, we created a heatmap to depict the distribution of immune cells. To further determine the relationship between PLAU expression and the immune cell proportions in each HNSCC sample, we constructed a correlation plot by correlation analysis. Furthermore, the Wilcoxon test and TIDE algorithm were used to compare the expression of eight immune checkpoint genes and the ICB response in HNSCC patients with high and low PLAU expression^23^.

### Statistical analysis

The majority of statistical studies were conducted by using the bioinformatics applications mentioned above, version 4.1.1 is used for all parts involving the R software. To examine the relative expression levels of RNAs, we utilized the two-tailed Student's t test and the Benjamini–Hochberg FDR technique for P value adjustment. The Spearman correlation coefficient analysis was performed to determine correlations. Only a p value of 0.05 was considered significant statistically.

## Results

### Identification of different immune genes

**Figure 1:**
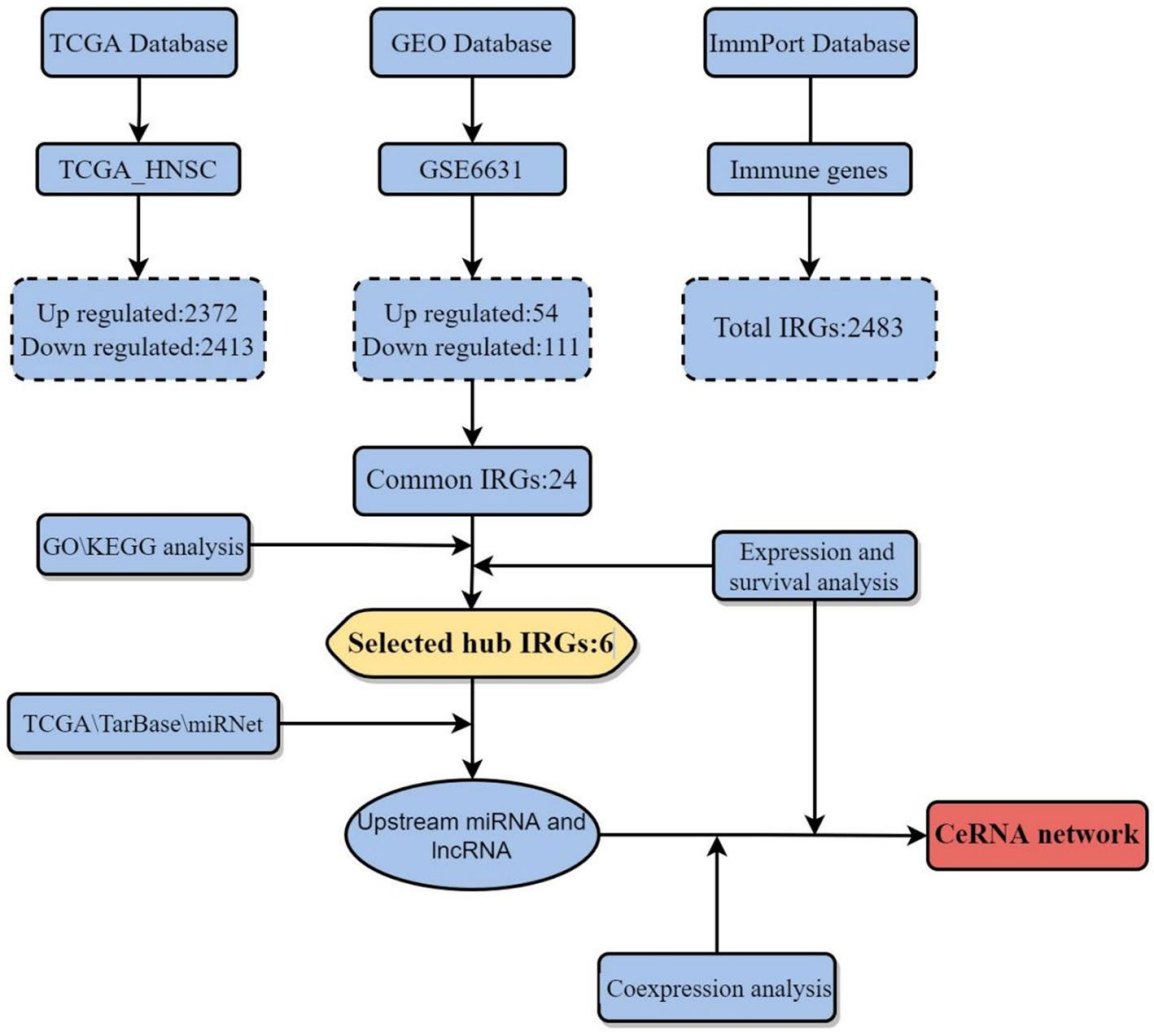
Flow chart of establishing and analyzing the immune-related ceRNA network.

**Figure 2:**
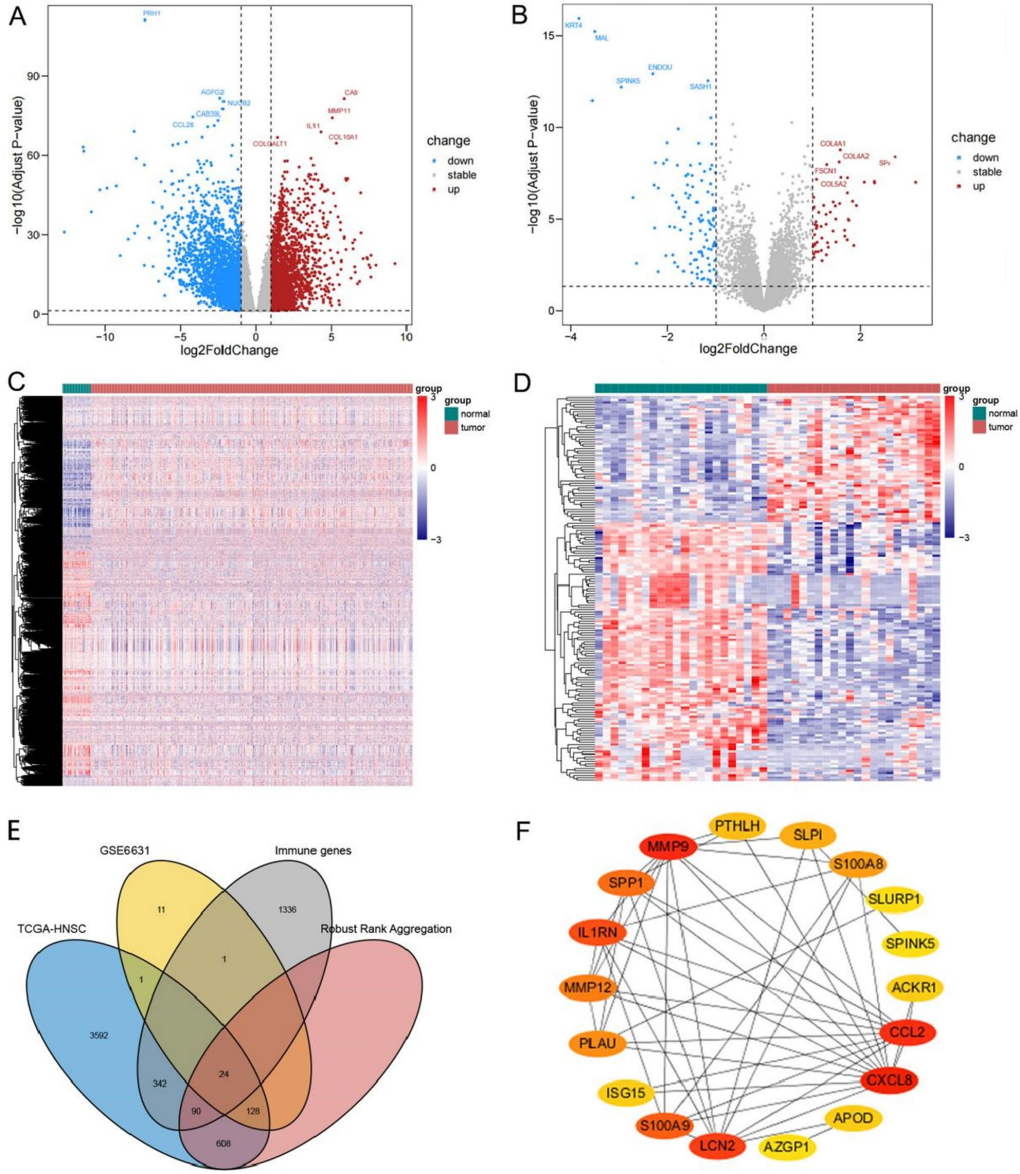
Assessment of differentially expressed genes (DEGs) in our analysis. (**A**, **B**) Volcano plots showing DEGs in TCGA-HNSC and GSE6631 (|log2 (Fold Change)| > 1, adjust p-value < 0.05); blue dots: significantly down-regulated; red dots: significantly up-regulated; grey dots: no significant differences; the top 10 significant genes are denoted. (**C**, **D**) Heatmaps showing DEGs of TCGA-HNSC and GSE6631. (**E**) Venn diagram showing detail information about overlaps of DEGs across four datasets. (**F**) The hub immune-related genes (IRGs) in our analysis.

The study flow chart and key goals are shown in Fig. 1. First, we identified DEGs via the TCGA and GEO databases. We incorporated genes that encode proteins from the TCGA-HNSC dataset, which includes 495 primary solid tumors and 44 normal samples, and 4785 DEGs (2413 upregulated and 2372 downregulated) were acquired. Overall, 165 DEGs were identified from GSE6631, including 54 downregulated genes and 111 upregulated genes (Fig. 2A–D). Finally, through rank analysis, 850 integrated DEGs, consisting of 347 upregulated genes and 503 downregulated genes, were identified by the RRA method, and 24 IRGs (Supplemental Table 1) were chosen for further analysis by taking the intersection of the common gene set and the IRGs downloaded from the ImmPort database (Fig. 2E).

### GO and KEGG enrichment analyses

**Figure 3:**
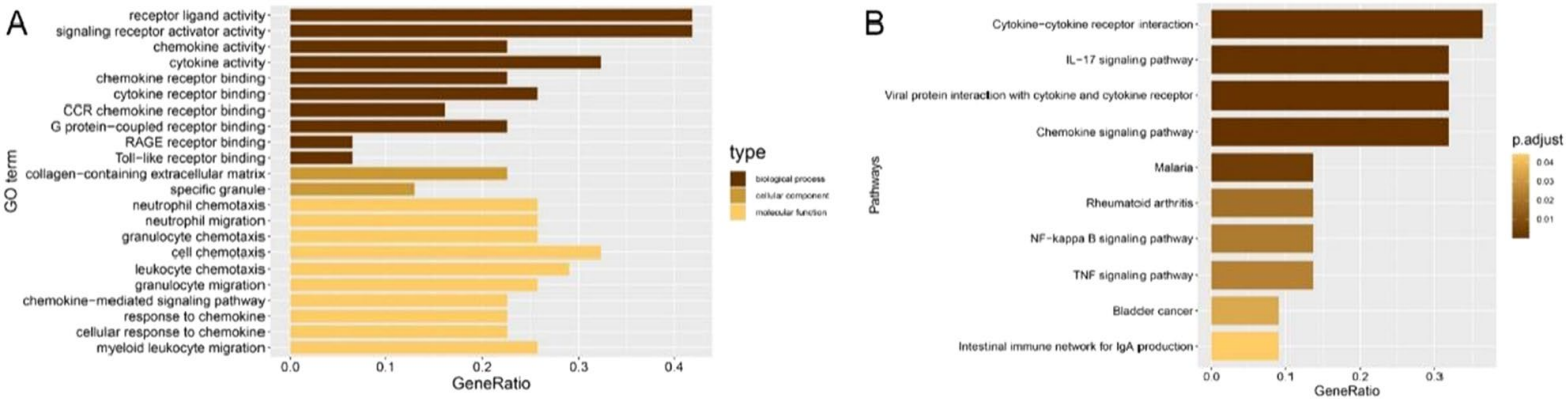
GO and KEGG pathway enrichment of IRGs. (**A**) GO enrichment; (**B**) KEGG pathway enrichment.

GO word and KEGG pathway analysis were used to speculate the biological function roles of the 24 is. There were 4 enriched terms in the cellular component (CC) category, 17 enriched terms in the biological process (BP), 2 enriched terms in the molecular function (MF) category, and 10 enriched KEGG pathways (Fig. 3A,B). Overall, receptor ligand activity was mainly mapped in the BP category, the collagen-containing extracellular matrix was enriched in the CC category, and cell chemotaxis was enriched in the MF category. In terms of KEGG pathways, we discovered that cytokine–cytokine receptor interactions, the IL-17 signaling route, and the chemokine signaling pathway were considerably enriched in the common important IRGs.

### Hub IRG identification and validation

**Figure 4:**
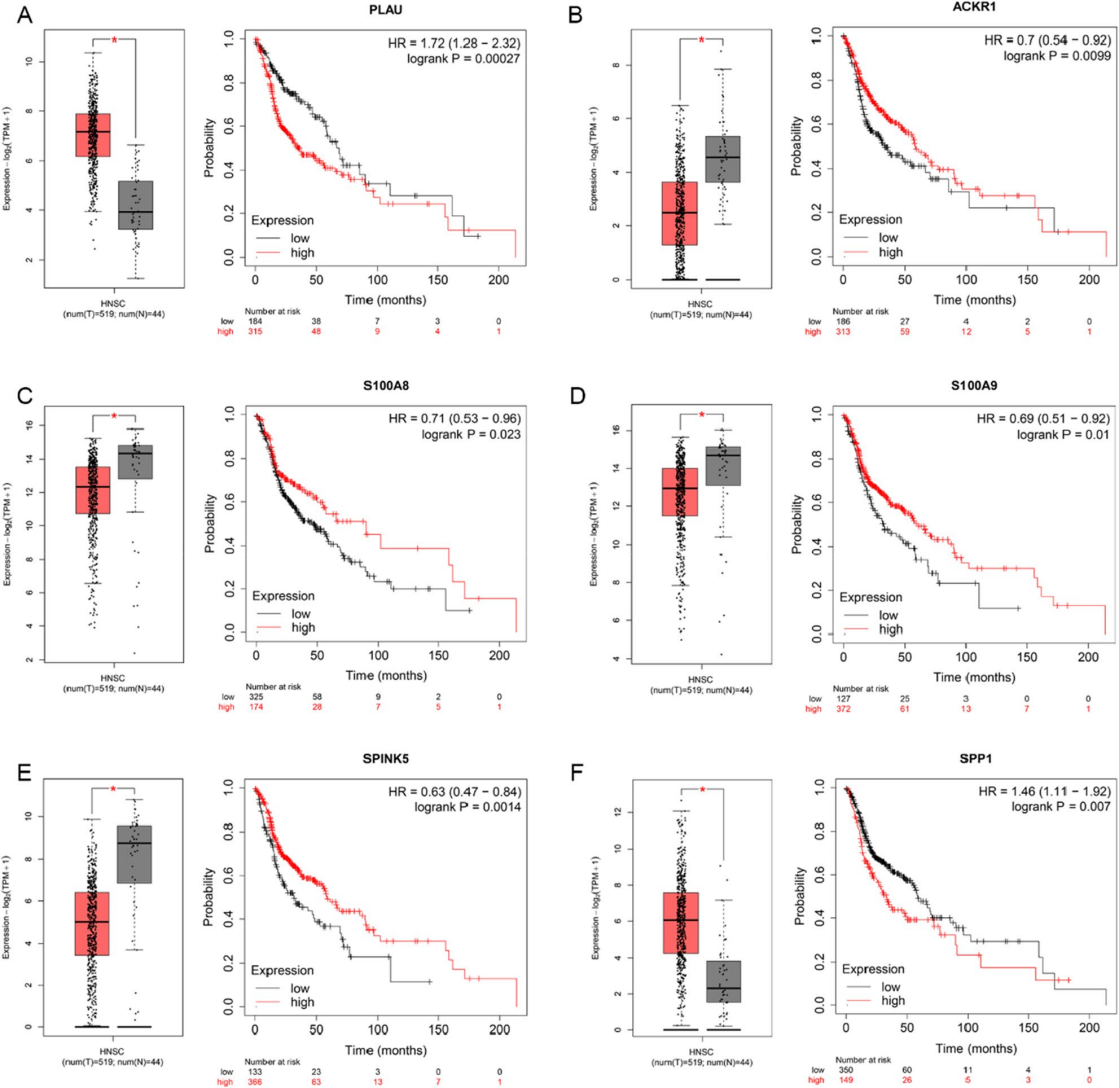
Hub IRGs expression and prognosis of the HNSCC patients. (**A**–**F**) Validation of PLUA, SPP1, S100A8, S100A9, SPINK5 and ACKR1 expression roles and prognosis values using GEPIA and KM plotter (*p < 0.05).

PPI networks were built to show the mutual interaction of the identified IRGs (Supplementary Fig. 1). We found 18 hub genes based on the node connectivity degree calculated by CytoHubba of Cytoscape software (Fig. 2F). Additionally, the KM plotter and GEPIA2 databases were used to determine the predictive significance of these hub genes in HNSCC patients. We found that 2 genes were upregulated (PLAU and SPP1) and 4 genes downregulated (S100A8, S100A9, SPINK5 and ACKR1), which significantly correlated with poor prognosis (p < 0.05) in HNSCC patients (Fig. 4A–F), and we selected these genes for further investigation.

### ceRNA network construction and survival analysis

**Figure 5:**
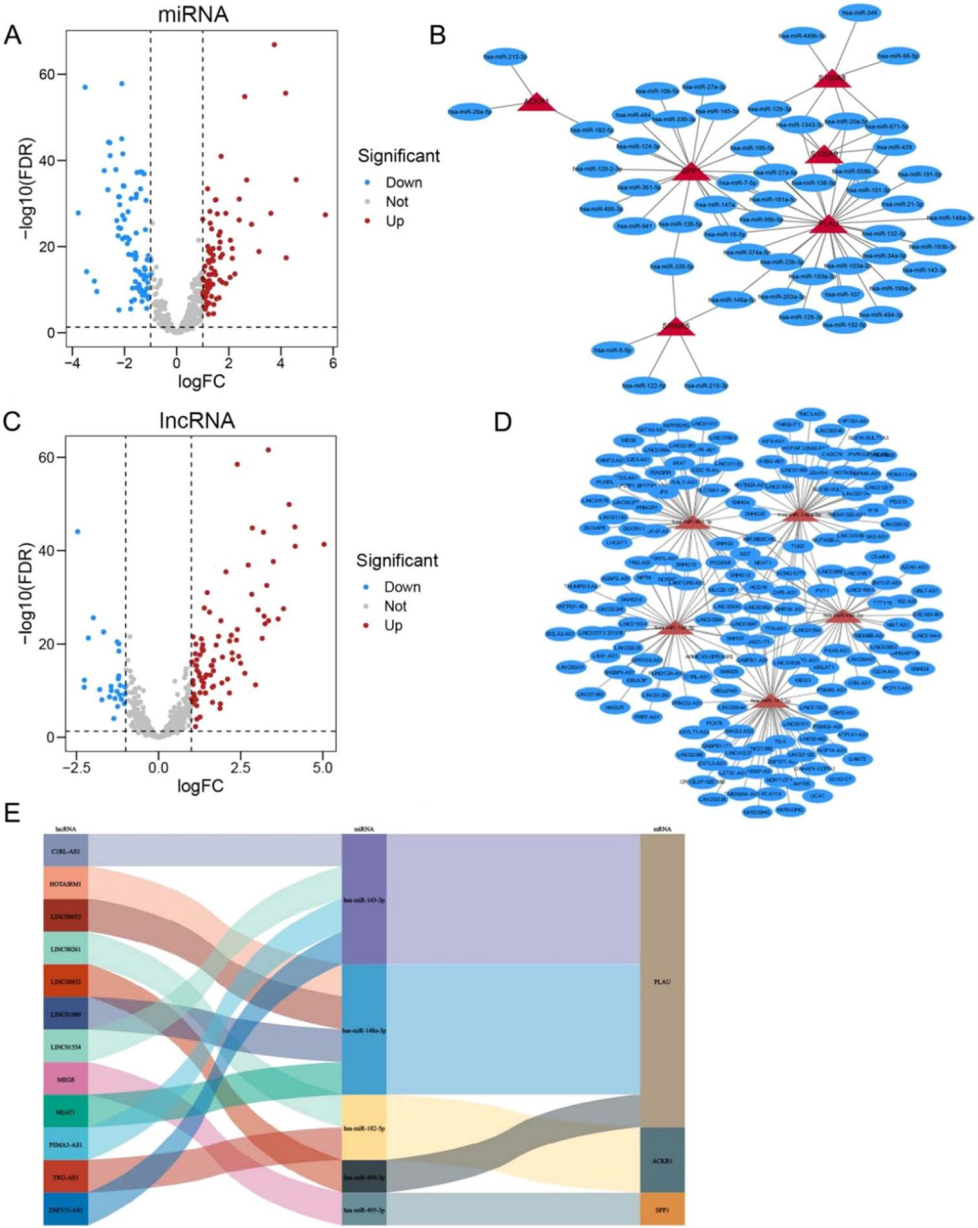
(**A**) The volcano plot of the differentially expressed miRNAs (|log (Fold Change)| > 0.5, adjust p-value < 0.05); blue dots: significantly down-regulated; red dots: significantly up-regulated; grey dots: no significant differences. (**B**) The mRNA-miRNA networks were identified using Tarbase and starBase; blue ellipse: miRNAs; red triangle: mRNAs. (**C**) The volcano plot of the differentially expressed lncRNAs (|log (Fold Change)| > 0.5, adjust p-value < 0.05); blue dots: significantly down-regulated; red dots: significantly up-regulated; grey dots: no significant differences. (**D**) The miRNA-lncRNA networks were identified using miRNet and starBase; blue ellipse: lncRNAs; red triangle: miRNAs. (**E**) Sankey diagram for the ceRNA network in HCC. Sankey diagram showing the potential lncRNA–miRNA–mRNA regulatory axes.

**Figure 6:**
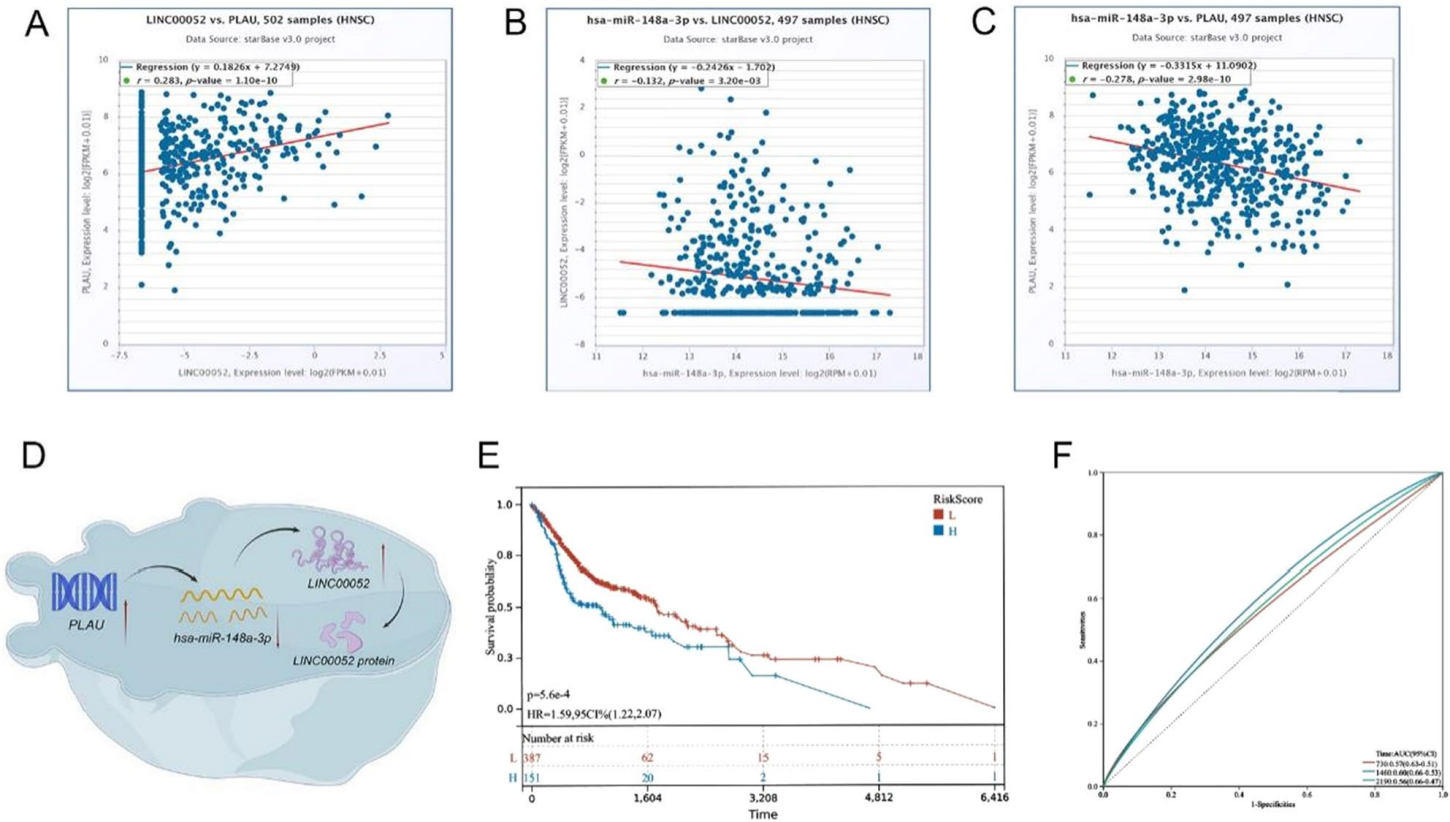
(**A**–**C**) Co-expression correlation analysis of qualified ceRNA network. (**D**) Schematic representations of the immune-related ceRNA network. (**E**) Kaplan–Meier survival curve by the risk score of the TCGA-HNSC dataset. (**F**) Time-dependent ROC curve analysis for survival prediction based on the risk score.

First, we identified 166 DEMs in the TCGA-HNSC dataset (Supplementary Table 2) and then visualized them by volcano (Fig. 5A). Then, miRNA–mRNA interactions were predicted with TarBase. After visualization of the data with Cytoscape, a total of 54 upstream miRNAs were selected with 6 IRGs, among which 75 miRNA–mRNA pairs were found (Fig. 5B). To further assess the expression and prognostic relevance of these miRNAs, we used starBase with a threshold of p value < 0.05. The above analysis identified 5 miRNAs that met the criteria and were included in the DEMs. Second, we identified DELs in TCGA-HNSC (Supplementary Table 3) and used the miRNet database to predict upstream lncRNAs (Fig. 5C,D). Additionally, similar to the above steps, 7 lncRNAs with significant (p < 0.05) differential expression and prognostic relevance were selected and added to the 5 miRNAs. As presented in Fig. 5E, we constructed a lncRNA–miRNA–mRNA network based on the above information. A qualifying lncRNA should be inversely linked with miRNA expression and favorably correlated with mRNA expression, according to the ceRNA hypothesis. As a result, we used co-expression analysis to filter all of the eligible lncRNA–miRNA and miRNA–mRNA pairs (Fig. 6A–C). The fledged ceRNA network (LINC00052/hsa-miR-148a-3p/PLAU) mechanism map is shown in Fig. 6D. Furthermore, we calculated the optimal truncation value of RiskScore and set the minimum sample size for grouping greater than 25% and the maximum sample size for grouping less than 75%. Finally, the optimal truncation value was obtained as follows: 0.0817416583277248. Based on this, the patients were divided into high and low groups to plot the Kaplan–Meier curves. Patients with high risk scores for HNSCC had less favorable clinical outcomes, as seen in Fig. 6E. In the meanwhile, ROC analysis of the TCGA dataset showed that the ceRNA network deduced the survival of KIRC patients at 2-year, 4-year, and 6-year survival rates (Fig. 6F).

### Pan-cancer analysis of LINC00052 expression and prognostic significance

**Figure 7:**
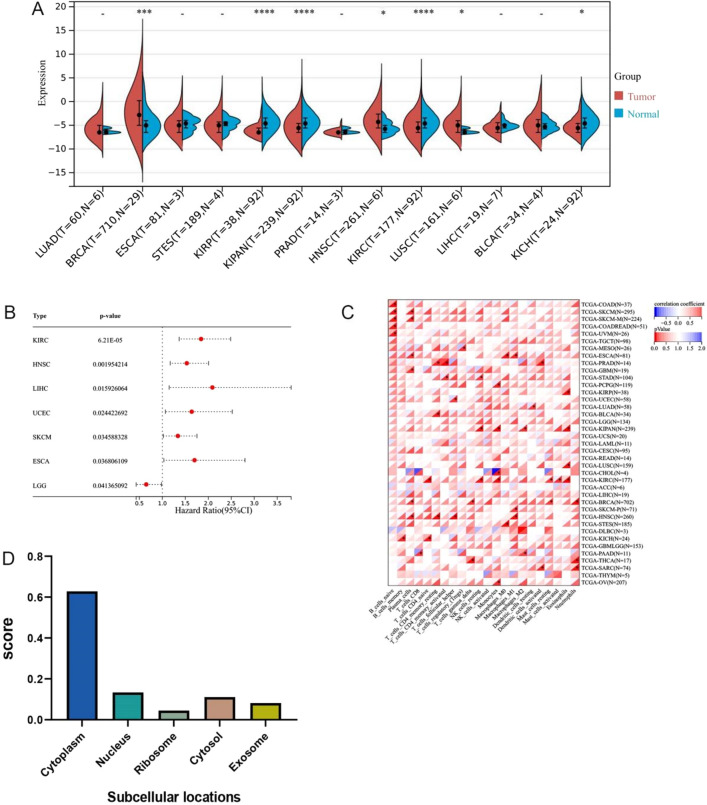
(**A**) The expression of LINC00052 in pan-cancer. (**B**) Forest plot of LINC00052 in multiple tumors was analyzed by univariate cox regression. (**C**) The correlation between expression of LINC00052 in different tumors and immune cell infiltration scores. (*p < 0.05) (**D**) The cellular localization for LINC00052 was predicted using lncLocator.

To evaluate the expression and prognostic significance of LINC00052 in various cancers, pan-cancer analysis was carried out based on TCGA data. We downloaded standardized data from the TCGA Pan-Cancer dataset (PANCAN, N = 10,535, G = 60,499) from UCSC Xena (https://xenabrowser.net/). The results showed that compared with normal tissues, 3 cancer types (breast invasive carcinoma (BRCA), lung squamous cell carcinoma (LUSC), and HNSCC) showed significantly increased and 4 cancer types (kidney renal papillary cell carcinoma (KIRP), pan-kidney cohort (KIPAN), kidney renal clear cell carcinoma (KIRC), and kidney chromophobe (KICH)) showed significantly decreased expression of LINC00052 according to R software analysis (Fig. 7A). The pan-cancer analysis showed that high expression of LINC00052 was associated with poor overall survival in 7 cancer types (KICH, HNSCC, LIHC, uterine corpus endometrial carcinoma (UCEC), skin cutaneous melanoma (SKCM), esophageal carcinoma (ESCA), and lower grade glioma (LGG)) according to univariate cox regression analysis (p < 0.05) (Fig. 7B). We assessed the invasion scores of 22 types of immune cells in 2949 tumor samples from 39 tumor types using the R software package CIBERSORT. Gene expression was significantly correlated with immune infiltration in 28 cancer types (glioblastoma multiforme (GBM), lung adenocarcinoma (LUAD), colon adenocarcinoma/rectum adenocarcinoma (COAD/READ), BRCA, ESCA, stomach and esophageal carcinoma (STES), sarcoma (SARC), KIRP, KIPAN, stomach adenocarcinoma (STAD), prostate adenocarcinoma (PRAD), UCEC, HNSCC, KIRC, LUSC, thyroid carcinoma (THCA), mesothelioma (MESO), metastatic skin cutaneous melanoma (SKCM-M), pancreatic adenocarcinoma (PAAD), ovarian serous cystadenocarcinoma (OV), pheochromocytoma and paraganglioma (PCPG), primary skin cutaneous melanoma (SKCM-P), uveal melanoma (UVM), bladder urothelial carcinoma (BLCA), KICH, cholangiocarcinoma (CHOL), skin cutaneous melanoma (SKCM), and testicular germ cell tumor (TGCT)) (Fig. 7C). Moreover, we determined where in the cell LINC00052 was most expressed by LncACTdb 3.0 (http://www.biobigdata.net/LncACTdb/). As shown in Fig. 7D, LINC00052 was mainly located in the cytoplasm.

### Relationship between the methylation and expression of PLAU

**Figure 8:**
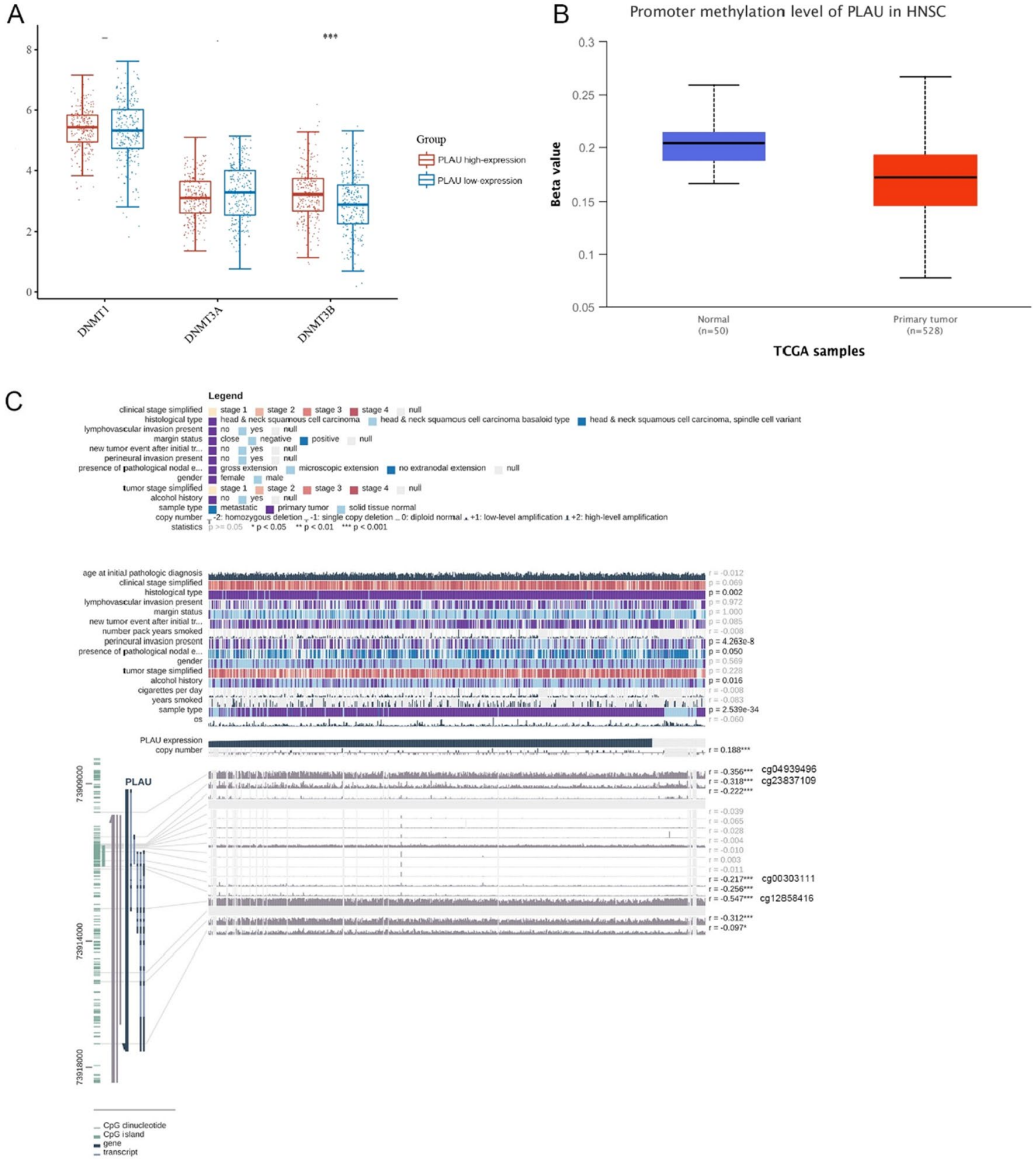
(**A**) Differential expression of three DNA methyltransferases (DNMT1, DNMT3A, and DNMT3B). (**B**) Methylation was evaluated using UALCAN. (**C**) The methylation site of PLAU DNA sequence association with gene expression was visualized using MEXPRESS. The top 4 most significant methylation sites are marked and were negatively correlated with PLAU expression. *p < 0.05, **p < 0.01, ***p < 0.001.

Growing evidence suggests that abnormal DNA methylation is an important characteristic of tumors and can be considered a biomarker to diagnose and predict the prognosis and occurrence of tumors^24^. Through analysis of DNA methyltransferase (DNMT1, DNMT3A, and DNMT3B) expression, we found that the key enzyme DNMT3B, which is involved in DNA methylation, was significantly upregulated in the high PLAU expression group (Fig. 8A). Furthermore, as shown in Fig. 8B, the methylation level of PLAU was notably lower in HNSCC tissues according to UALCAN analysis. Subsequently, four methylation sites in PLAU DNA sequences that were negatively linked with expression levels were identified (cg04939496, cg23837109, cg00303111, and cg12858416) (Fig. 8C).

### Immune landscape in HNSCC

**Figure 9:**
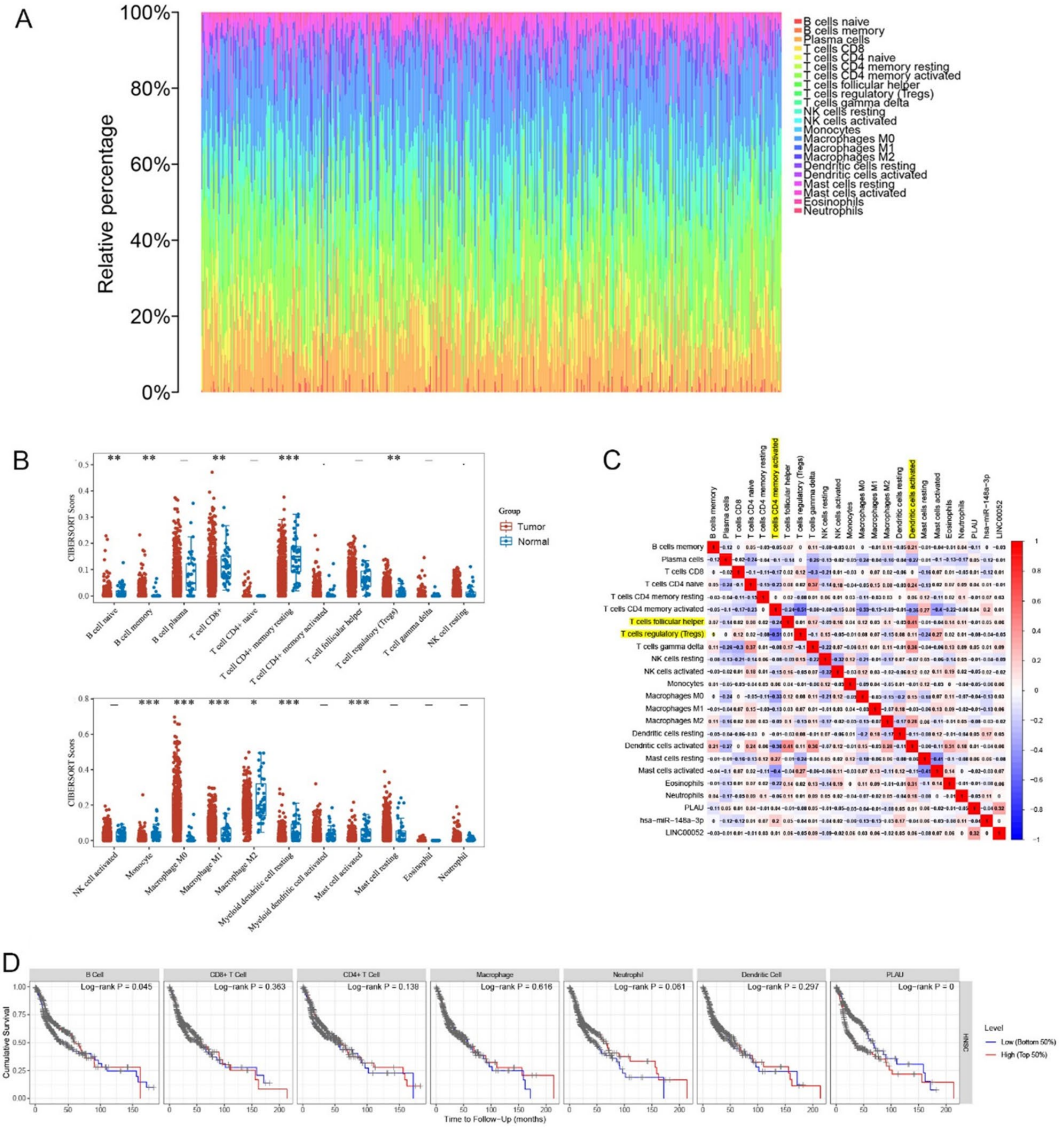
Analysis of immune infiltration. (**A**) The composition of 22 immune cells was estimated by CIBERSORT in HNSCC. (**B**) Difference in the proportions of 22 immune cells between normal and tumor tissues (*p < 0.05, **p < 0.01, ***p < 0.001). (**C**) The correlation index among immune cells and ceRNA in HNSCC. Positive and negative correlations are represented by the red and blue colors, respectively. The degree of correlation index is represented by the color depth. The yellow color shows the genes with the highest positive and negative correlations. (**D**) Kaplan–Meier plots were used to analyze the immune infiltration and overall survival rate of HNSCC.

Immunotherapy mainly targets the tumor immune microenvironment. CIBERSORT analysis of RNA-seq data from HNSCC was used to reveal the proportions of immune cells in each sample (Fig. 9A). The violin chart showed 11 kinds of immune cells (B cell naive, B cell memory, T cell CD8+, T cell CD4+ memory resting, T cell regulatory (Tregs), Monocyte, macrophages M0/ M1/M2, Myeloid dendritic cell resting, activated Mast cells) that were present in significantly different proportions in HNSCC tissues (Fig. 9B). It was also determined that tumor-infiltrating immune cells in HNSCC had a strong correlation. In all samples, there was a substantial negative association between Tregs and activated memory CD4 + T cells (R = 0.51), while a significant positive correlation between follicular helper T cells and activated dendritic cells (R = − 0.40) (Fig. 9C). However, only B cells were found to be a significant predictor of the outcome of HNSCC (Fig. 9D).

### High PLAU expression may be related to the tumor immune response

**Figure 10:**
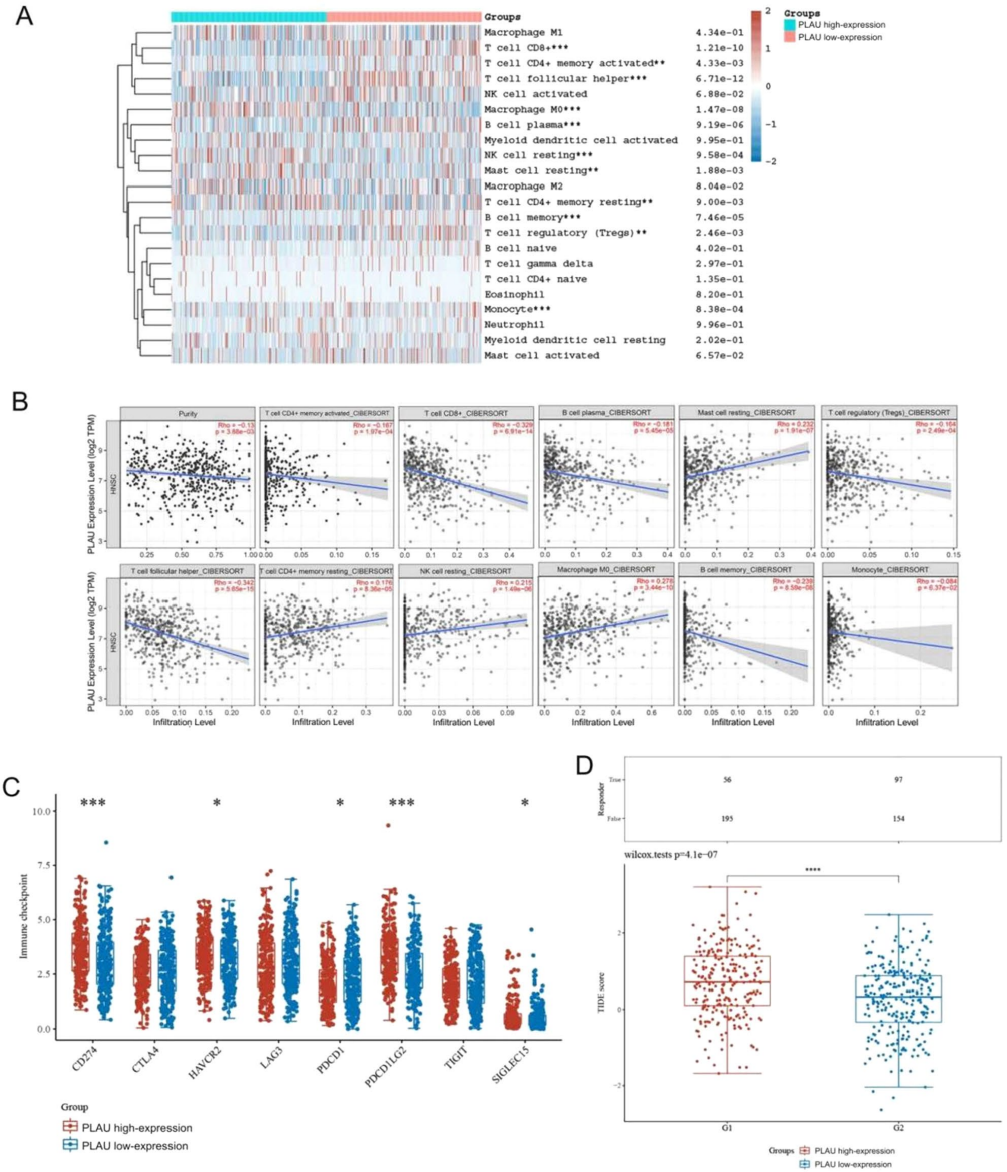
(**A**) Immune cell score heatmap, clustered by their relative expression of the PLAU. (**B**) Correlation between PLAU expression and immune infiltration levels. (**C**) The distribution of 8 immune checkpoint genes’ expression in high- and low-PLAU expression of HNSCC. (**D**) TIDE scores between high- and low-PLAU expression of HNSCC. *p < 0.05, **p < 0.01, ***p < 0.001.

A heatmap of the CIBERSORT analysis results showed that PLAU was correlated with 11 immune cell types (Fig. 10A). The infiltration of T cells CD8+, T cells CD4+ memory activated, T cells follicular helper, B cell plasma, B cell memory, and Tregs was inversely related to PLAU expression, while T cells CD4+ memory resting, macrophage M0, mast cells resting, and NK cells resting were positively related to PLAU expression (Fig. 10B). These findings further demonstrated that PLAU expression has a major impact on the proportions of immune cells in the HNSCC immune microenvironment. Immune checkpoint molecules are inhibitory regulatory molecules in the immune system, and their expression can impair immune cell activity such that a tumor can evade immune elimination. We assessed the correlation between PLAU levels and immune checkpoint levels to determine whether we could predict immunotherapy response using target genes in the ceRNA subnetwork. We discovered that in samples with high PLAU expression, the expression of four of eight immunosuppressive factors was increased, with CD274 and PDCD1LG2 showing the largest increase (Fig. 10C). The TIDE algorithm was then used to predict immunotherapy response. A high likelihood of responding to ICB therapy is suggested by a low TIDE score. As shown in Fig. 10D, HNSCC patients with lower TIDE scores were more likely to be in the low PLAU expression group. In summary, HNSCC patients with low PLAU expression are more likely to benefit from immune checkpoint inhibitors. This phenomenon may occur because the key genes in our ceRNA prognostic subnetwork regulate some types of immune cells and the expression of immune checkpoints in the tumor microenvironment.

## Discussion

HNSCC is a solid tumor that has high immune infiltration, aggressiveness and heterogeneity^25,26^. HNSCC has conventionally been treated with surgery, radiotherapy, and chemotherapy. However, these therapies do not generate satisfactory results, and patients are in desperate need of novel therapies to improve their prognosis. Immunotherapy is a relatively recent and popular treatment option^27^. HNSCC is a heterogeneous disease, featuring differences in expression profiles, immune infiltration, and mutations, implying that immunotherapies could be successful^28^. Therefore, studying the molecular mechanisms and processes underlying HNSCC development and identifying appropriate biomarkers are critical for developing new treatment targets and improving patient outcomes.

In recent years, RNA molecules have attracted much attention due to their diverse functions and crucial roles in virtually every metabolic pathway and process. On the other hand, their ability to establish intricate networks of interactions makes it difficult to comprehend and characterize their modes of action. ceRNA networks are newly recognized regulatory mechanisms implicated in the initiation and progression of human malignancies that are being rapidly explored by numerous preclinical and clinical studies^29^. Earlier research into ceRNAs in HNSCC found many RNAs linked to patients OS^30–32^. Guo et al. constructed two risk signatures on nine ceRNAs (ANLN, CFL2, ITGA5, KDELC1, KIF23, NFIA, PTX3, RELT, and TMC7) and three immune cell types (naïve B cells, neutrophils, and regulatory T cells)^9^. These immune cells also showed a correlation with our ceRNAs. These studies clarified how ceRNAs can improve the accuracy of HNSCC diagnosis and prognosis evaluation. Unlike other studies that screened DEGs from the TCGA or GEO and then performed immune infiltration analysis, our study combined the TCGA, GEO and ImmPort databases to screen the IRGs. Then we obtained the upstream miRNAs and lncRNAs to construct an immune-related ceRNA network, which was verified by comprehensive biological analysis. Finally, a prognostic regulatory axis (LINC00052/hsa-miR-148a-3p/PLAU) was found. Our study builds upon previous studies and reveals the relationship between the key genes in this axis and DNA methylation, immune infiltration and immune checkpoints.

Evidence accumulated over the past decade shows that lncRNAs are widely expressed and have a significant impact on a variety of physiological and pathological processes, and their potential role as biomarkers has been widely discussed. Indeed, increased levels of LINC00052 are implicated in the formation, development, and metastasis of a variety of cancers^33,34^. Likewise, in HNSCC, a recent study indicated that LINC00052 sponged miR-608 to control epidermal growth factor receptor (EGFR) expression, thereby encouraging advancement both in vitro and in vivo^35^. Upon further research, we found that LINC00052 was mostly located in the cytoplasm and highly expressed in HNSCC tissues with poor prognosis, in line with past studies. hsa-miR-148a-3p, whose family has already been proven to be biomarkers closely related to hepatocellular carcinoma (HCC)^36^, colon adenocarcinoma (COAD)^37^, acute myeloid leukemia (AML)^38^, esophageal squamous cell carcinoma (ESCC)^39^ and gastric cancer (GC)^40^, is directly linked to vascular invasion. Ashizawa et al. indicated that Overexpression of miR-148a-3p in colorectal cancer reversed IFN-induced PD-L1 expression on the tumor cell surface, which was functionally connected to lower T-cell apoptosis in the TME^41^. Simultaneously, Zhou et al.^42^ showed that low hsa-miR-148a-3p expression was linked to naive B cells and poor prognosis in HNSCC and that hsa-miR-148a-3p may operate like a sponge to modify PI3K/Akt signaling. Our findings support previous research that hsa-miR-148a-3p has tumor-suppressing properties.

PLAU encodes a serine protease that can convert plasminogen to plasmin, degrade the tumor-surrounding stroma and promote the migration of cells to distant organs, resulting in tumor cell invasion, migration, and homing to distant organs^43^. To date, numerous investigations have established that PLAU was significantly enhanced and participated in the metastasis of HNSCC^44–46^, but its related ceRNAs have not been described in HNSCC. Many human malignancies, including HNSCC, are influenced by abnormal DNA methylation, particularly promoter hypermethylation and methylation inducing transcriptional suppression of tumor suppressor genes^47^. In our study, we discovered that in HNSCC tissues, the level of PLAU methylation was much lower than that in normal tissues, implying that low DNA methylation promoted PLAU expression. Furthermore, aberrant methylation of cg04939496, cg23837109, cg00303111, and cg12858416, which mainly fell in the untranslated region (UTR) and TSS1500 region, resulted in the upregulation of PLAU in HNSCC.

Immune infiltration in the TME is a complex process that involves numerous subsets of lymphocytes infiltrating tumors and interacting with tumor cells and/or with one another via diverse networks^48^. Cancer growth, angiogenesis, and metastasis are all affected by immune cells that infiltrate the tumor, according to numerous studies. A combination of tumor-infiltrating lymphocytes (T lymphocytes, B lymphocytes, and natural killer (NK) cells) and cells of the myeloid lineage (macrophages, neutrophils, dendritic cells, and MDSCs) makes up the immunological component of the HNSCC TME^1^. In earlier research, 56% of HNSCC patients showed reactivity against at least one tumor-associated antigen (TAA). Patients with TAA responses had a higher percentage of CD4+ T cells infiltrating their tumors, compared to tumors with no TAA response, which predominantly had CD8+ T cells^49^. B cells play an important role in the adaptive immune system, responding to infected cells or tumor cells and regulating cellular immune responses both positively and negatively^50^. By using the CIBERSORT algorithm, we observed that 11 types of immune cells showed significantly different abundances in HNSCC (p < 0.05). Survival analysis showed that only B cells were associated with a positive prognosis in HNSCC. The findings of our research matched those of the prior experiments. PLAU was chosen for further investigation. In the above tests, we consistently observed a higher expression of PLAU associated with poor outcomes in HNSCC. Another important finding of this study is that PLAU expression was significantly associated with the infiltration of diverse immune cells in HNSCC. We found that PLAU is important for regulating immune cell infiltration in HNSCC, with particularly strong negative effects on CD8+ T cells, memory activated CD4+ T cells, follicular helper T cells, plasma B cells, memory B cells, and Tregs and positive effects on memory resting CD4+ T cells, M0 macrophages, resting mast cells, and NK cells resting. Numerous studies have revealed that T cells CD8+, Tregs, B cells naïve, T cells follicular helper, and T cells CD4 memory activated were associated with improved outcomes in HNSCC patients. In addition, in a pan-cancer analysis, high PLAU expression was consistently associated with monocyte infiltration in the TME^51^. These studies demonstrate that PLAU may mediate the proportions of infiltrating immune cells, indirectly leading to immune escape.

Immunological checkpoints, as regulatory signals, play a critical role in maintaining self-tolerance by modulating T-cell function and defending host tissues against indirect immune-mediated damage; therefore, tumors can evade attack by immune cells^7^. Immune checkpoint inhibitors (ICIs), such as those targeting PD-L1, PD-1, and cytotoxic T lymphocyte-associated antigen-4 (CTLA-4), have been investigated across multiple tumor types and have shown significant benefits in clinical studies^10^. ICIs, on the other hand, have limitations in HNSCC patients. The efficiency of ICIs is determined by a variety of factors, including the immunological TME, and only a few indicators correctly predict patient prognosis. It is necessary for us to find reliable predictive biomarkers. Certain biomarkers, including those found by TIDE, are more accurate at predicting the fate of melanoma patients treated with first-line anti-PD-1 or anti-CTLA-4 antibodies than other biomarkers, such as PD-L1 levels and tumor mutation burden (TMB)^23^. We discovered that increased PLAU expression in HNSCC patients may be associated with resistance to immune checkpoint blockade therapy by TIDE analysis. In a pan-cancer study, high PLAU expression was consistently associated with important immune response checkpoints such as PD-L2 and CD276/B7-H3^51^. In our investigation, we discovered a strong correlation between high PLAU expression and important checkpoints, such as CD274 and PDCD1LG2. PD-L1 and PD-L2 are immunosuppressive proteins encoded by CD274 and PDCD1LG2, respectively, that restrict CD4+ and CD8+ T lymphocytes from generating cytokines and engaging in cytolytic activity^52,53^. Monoclonal antibodies directed against PD-L1 and PD-L2 are currently being utilized in clinical trials for patients with advanced solid tumors with FDA permission^54^. The results of this study could further explain the mechanism underlying the relationship between poor prognosis and high PLAU expression in HNSCC.

The study and its findings are limited by several factors. First, this study relied on bioinformatics analysis and did not use clinical samples to test the signature's accuracy. It is still unclear how these genes are regulated in HNSCC, and further study will be needed to determine their biological functions both in vitro and in vivo.

## Conclusion

In conclusion, we constructed a prognosis-related ceRNA network (LINC00052/hsa-miR-148a-3p/PLAU) based on IRGs, which we then verified using several approaches. This network can be used to assess the prognosis of patients with HNSCC and also to predict their responsiveness to ICI therapy.

## Supplementary Information

Supplementary Legends.

Supplementary Figure 1.

Supplementary Figure 2.

Supplementary Figure 3.

Supplementary Table 1.

Supplementary Table 2.

Supplementary Table 3.

## Data Availability

The original contributions presented in the study are included in the article/Supplementary Material, further inquiries can be directed to the corresponding author/s.
