# Supplementary material for: Identification of a novel ceRNA network related to prognosis and immunity in HNSCC based on integrated bioinformatic investigation
Source: Sci Rep. 2022 Oct 20;12:17560. doi: 10.1038/s41598-022-21473-0 (PMC9584951; doi:10.1038/s41598-022-21473-0)
Supplement: Supplementary file 1 — Supplementary Legends. [file 41598_2022_21473_MOESM1_ESM.docx]

**Supplementary Material**

Supplementary Figure 1. PPI network analysis of IRGs in the STRING database.

Supplementary Figure 2. The expression level and predictive value of potential miRNAs in HNSCC are being screened and validated. (A–E) High levels of hsa-miR-494-3p, hsa-miR-143-3p, and hsa-miR-495-3p were linked to a poor prognosis, while low levels of hsa-miR-182-5p and hsa-miR-148-3p were linked to prolonged survival.

Supplementary Figure 3. The expression level and predictive value of potential miRNAs in HNSCC are being screened and validated. (A–L) High expression of HOTAIRM1, LINC00261, MEG8, and LINC00052 were related to a bleak prognosis, whereas low expression of C1RL-AS1, LINC00852, PSMA3-AS1, LINC01089, TRG-AS1, ZNFS71-AS1, NEAT1, and LINC01554 were associated with dismal survival.

Supplementary Table 1. Different expression genes (DEGs) and common immune-related genes (IRGs) in our analysis.

Supplementary Table 2. Different expression miRNAs (DEMs) in our analysis.

Supplementary Table 3. Different expression lncRNAs (DELs) in our analysis.
