# Supplementary figures and images for: Identification of a novel ceRNA network related to prognosis and immunity in HNSCC based on integrated bioinformatic investigation
Source: Sci Rep. 2022 Oct 20;12:17560. doi: 10.1038/s41598-022-21473-0 (PMC9584951; doi:10.1038/s41598-022-21473-0)

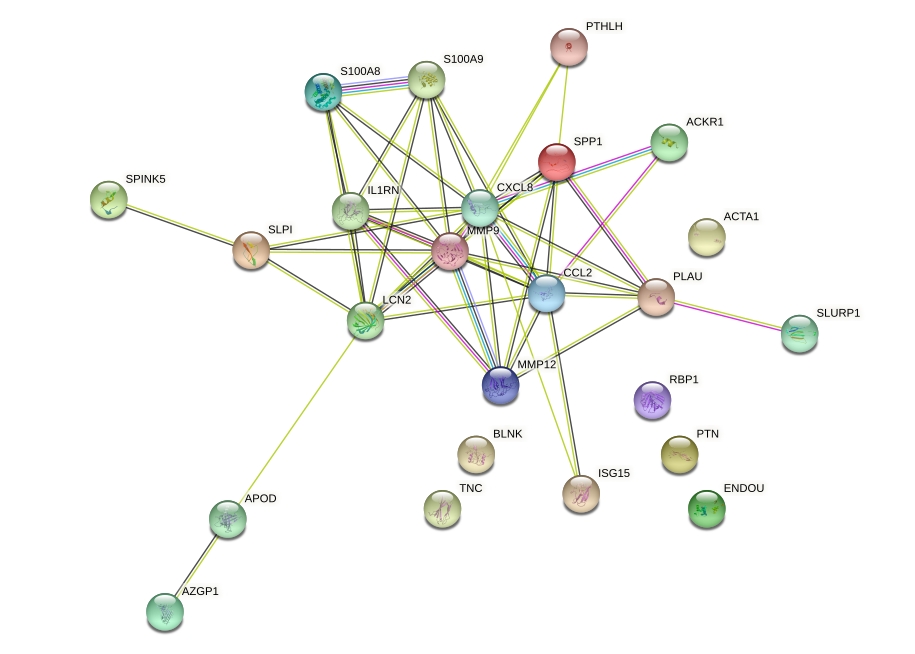

Supplement: Supplementary file 2 — Supplementary Figure 1. [file 41598_2022_21473_MOESM2_ESM.jpg]

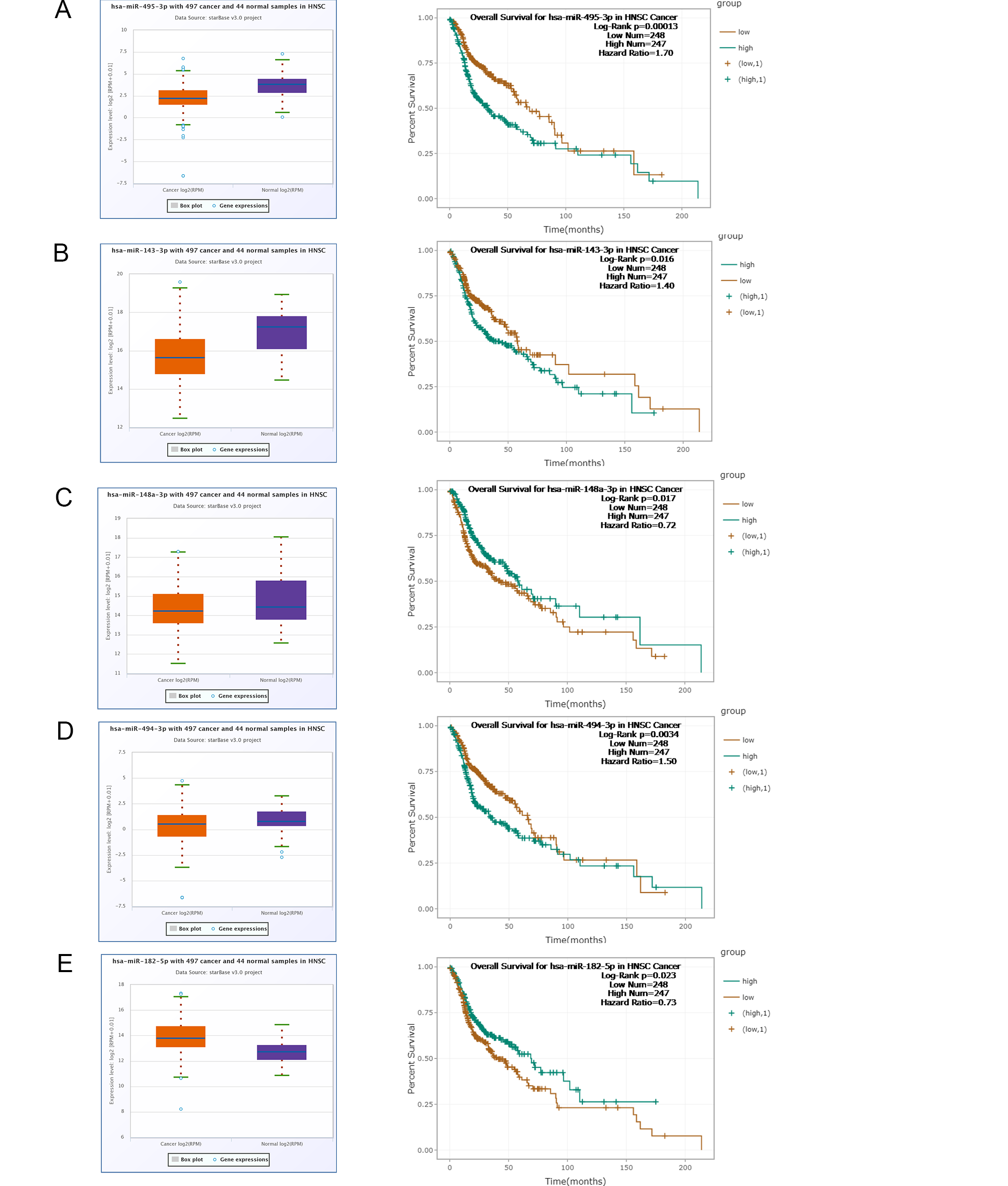

Supplement: Supplementary file 3 — Supplementary Figure 2. [file 41598_2022_21473_MOESM3_ESM.jpg]

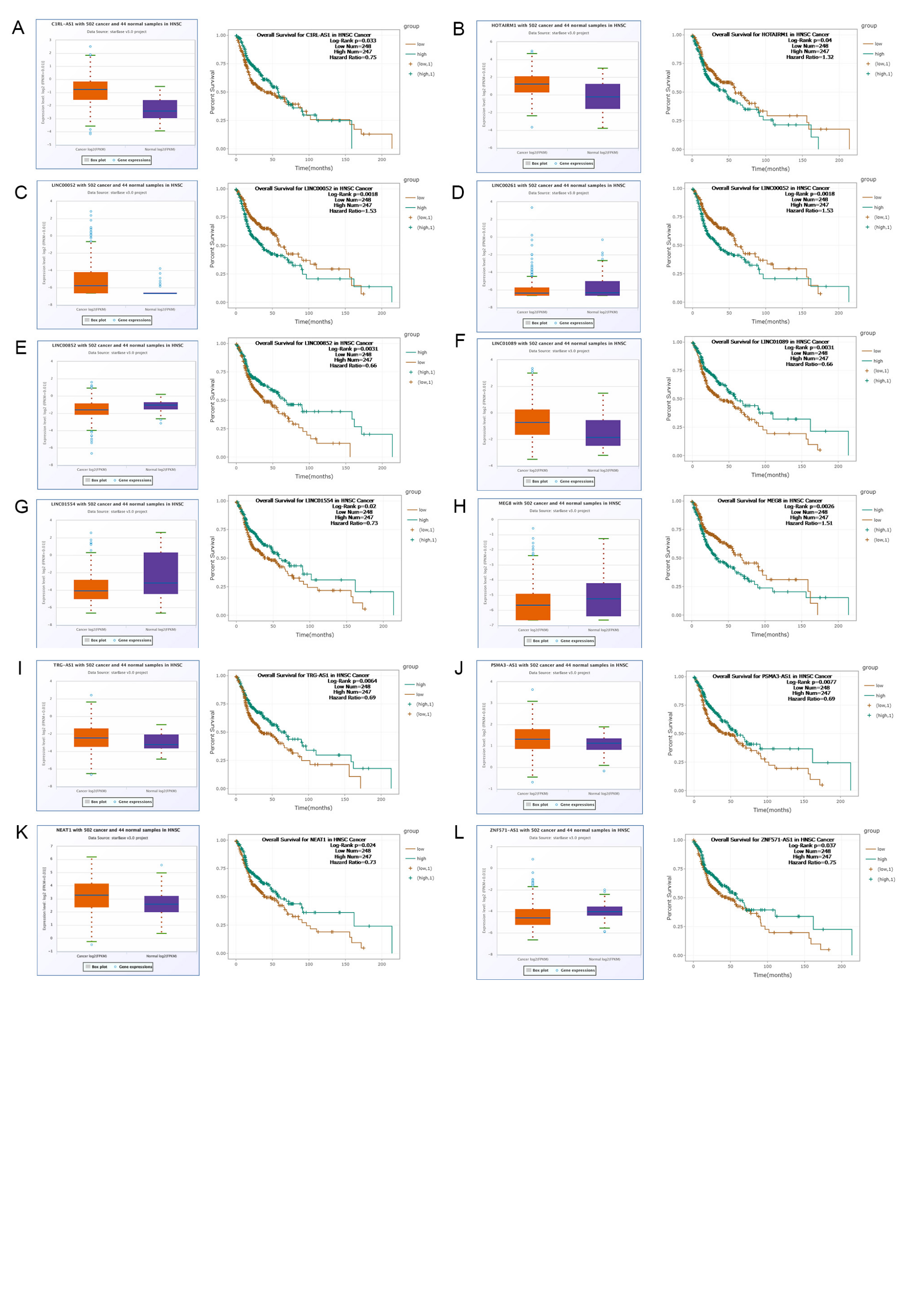

Supplement: Supplementary file 4 — Supplementary Figure 3. [file 41598_2022_21473_MOESM4_ESM.jpg]
